# Can You Determine the Cause of This Patient’s Skin Changes?

**Published:** 2012-09-01

**Authors:** Sandra Kurtin

**Affiliations:** From Arizona Cancer Center, Tucson, Arizona

**History**

Mr. M. is an 80-year-old African American male with a long-standing history of low-back pain, urinary incontinence, and intermittent stool incontinence following external beam radiation for treatment of stage T1C prostate cancer. Additional medical history includes hypertension and diabetes with existing peripheral neuropathy. He developed progressive low-back pain with radiation to the right buttocks and posterior thigh. A CT of the pelvis revealed an S3 sacral meningocele, extending anteriorly, inferiorly, and posteriorly, with the largest dimensions being approximately 10.2 × 11.0 cm. He underwent laparoscopic loop colostomy followed by resection of the meningocele. Pathology demonstrated a pooly differentiated adenocarcinoma consistent with a rectosigmoid primary, with tumor present at the distal surgical margins.

Mr. M. was referred to medical oncology for newly diagnosed locally advanced poorly differentiated mucinous rectosigmoid adenocarcinoma. He received palliative chemoradiotherapy to a residual right gluteal mass for pain control using oral capecitabine 1,000 mg bid (Monday through Friday) concurrent with 4,500 cGy over 20 fractions (4 weeks), with improvement in his level of pain. The treatment was tolerated with mild diarrhea. Given Mr. M.’s performance status (Eastern Cooperative Oncology Group 1/2) and comorbidities, he then continued palliative chemotherapy with oral capecitabine 1,500 mg bid days 1–14 every 21 days.

## Chief Complaint

**Figure 1:**
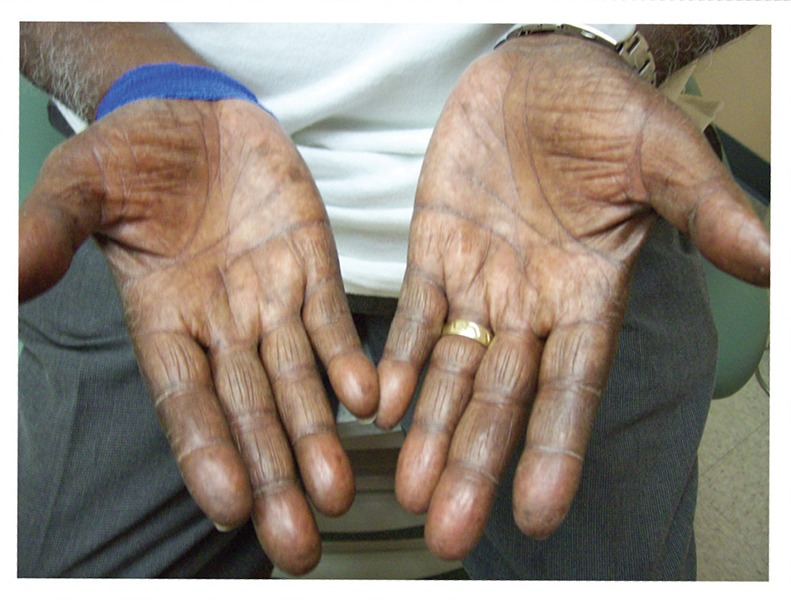
Figure 1. Diagnostic Snapshot

Mr. M. presented for an unscheduled visit on day 17 of cycle 2 of single-agent capecitabine. He reported numerous black spots on his face, hands, and feet associated with pruritus (Figure 1). He also reported increased numbness and tingling in his feet, persistent mild diarrhea with increased irritation of the stoma, similar black spots on the stoma, and increased fatigue. He denied any bleeding from the stoma and did not report any oral tenderness. He did report mild nausea without emesis.

## Physical Examination and Diagnostic Studies

On physical exam, Mr. M. appeared fatigued. Vital signs were normal. Multiple areas of painless hyperpigmentation were noted on the palmar and plantar surfaces, the face, tongue, buccal mucosa, nail beds, and the exposed stoma. No ulcerations or exudate were noted. The palmar surfaces were noted to be leathery, with mild tenderness and fullness of the fingertips. Xerosis was noted on the sun-exposed areas of the skin with some flaking. Laboratory analysis revealed a normal complete blood count, potassium 3.0 mMol/L (normal: 3.5–5.5), otherwise normal complete metabolic panel, and stool negative for *Clostridium difficile* or blood.

## Choose the correct diagnosis:

- DIHYDROPYRIMIDINE DEHYDROGENASE (DPD) DEFICIENCY
- STEVENS-JOHNSON SYNDROME
- CAPECITABINE-ASSOCIATED HYPERPIGMENTATION AND PALMAR-PLANTAR ERYTHRODYSESTHESIA (PPE)

**Scroll down for correct answer.** 

## Correct Answer 

**Capecitabine-associated hyperpigmentation and palmar-plantar erythrodysesthesia.** Capecitabine is an orally administered prodrug of fluorouracil (5-FU) commonly used alone or in combination for adjuvant treatment of resected colorectal cancer and in the treatment of metastatic colorectal or metastatic breast cancer (Roche, 2005). The most common adverse events seen in clinical trials using 5-FU and capecitabine are similar, including cytopenias, diarrhea, nausea, and PPE or hand-foot syndrome (Silvestris et al., 2010; Roche, 2005) The incidence of PPE is more common in capecitabine-containing regimens when compared with 5-FU, with the incidence of grade 3 PPE in phase III trials reported in 16.2%–18% for capecitabine and 0.3%–1% for 5-FU–based regimens (Silvestris et al., 2010).

The National Cancer Institute (NCI) Common Terminology Criteria for Adverse Events (CTCAE) V4.0 defines grade 3 PPE as the presence of severe skin changes (peeling, blisters, bleeding, edema, or hyperkeratosis) with pain, limiting activities of daily living (NCI, 2010). The underlying physiologic process for the development of PPE is poorly understood, although drug accumulation in the eccrine (sweat) glands of the hands and feet, the increased concentration of thymidine phosphorylase (activates capecitabine) on the palmar and plantar surfaces, and local irritation or friction to these areas with secondary capillary damage during daily activities are thought to play a role. The onset of PPE is most common after 6 weeks of therapy and dose dependent in the majority of cases (Saif et al., 2007; Gressett, Stanford, & Hardwicke, 2006; Silvestris et al., 2010).

Hyperpigmentation resulting from capecitabine and 5-FU has not been widely reported in clinical trials and is not included in the symptoms associated with PPE, yet it has been frequently observed in clinical practice. Case reports in the literature suggest an increased incidence in nonwhite patients and a correlation with impending onset of grade 2 or 3 PPE (Saif & Sandoval, 2008; Vickers & Easaw, 2008). Capecitabine-induced hyperpigmentation is most common in sun-exposed regions, palmar and plantar surfaces, nail beds, oral mucosa, and in the case of this patient, the exposed stoma. The physiology of hyperpigmentation is not well understood; however, these changes generally resolve within 3 to 6 months following discontinuation of capecitabine.

## Explanation of Incorrect Answers

**Dihydropyrimidine dehydrogenase (DPD) deficiency.** Deficiency of the DPD enzyme is a relatively rare (3%–5% of cancer patients) autosomal recessive disorder resulting in the ineffective metabolism of 5-FU and capecitabine (Saif, Ezzeldin, Vance, Sellers, & Diasio, 2007). Patients with heterozygous mutation in the *DPYD* gene are most susceptible to severe and potentially life-threatening toxicities with standard doses of fluoropyrimidine agents due to the accumulation and prolonged exposure to toxic compounds (Saif et al., 2007). Symptoms most often develop within the first 7 to 14 days of exposure and include grade 3/4 neutropenia, grade 3/4 mucositis, cerebellar dysfunction, diarrhea, and death in up to 100% of patients with complete DPD deficiency (very rare) (Moore, 2009). Early onset of grade 3 PPE in patients treated with capecitabine may be due to DPD deficiency.

**Stevens-Johnson syndrome** is a variant of an epidermolytic adverse cutaneous reaction characterized by a prodrome of flu-like symptoms (fever, myalgia) followed by rapid onset and coalescence of mucosal erosions and target lesions (central ulceration surrounded by pale erythematous rings) with eventual sloughing of < 10% of the superficial layer of involved skin (Harr & French, 2010). If more than 10% of the skin surface is involved with similar findings, the patient is considered to have toxic epidermal necrolysis, a more severe and in many cases life-threatening variant. Drug reactions are the most common cause, with onset between 7 and 60 days of exposure (Harr & French, 2010).

## Management

Early identification, dose delays, and/or dose modification of capecitabine remain the most effective strategies for management of PPE (Gressett et al., 2006; Silvestris et al., 2010). Dose modification is not necessary for hyperpigmentation. Dose adjustment of capecitabine for renal insufficiency (creatinine clearance between 30–50 mL/min) is necessary, and the use of capecitabine in patients with a creatinine clearance of < 30 mL/min is not recommended (Roche, 2005). Supportive care measures should be initiated at the start of capecitabine therapy to reduce the severity of PPE: applying alcohol-free emollient lotions to the palmar and plantar surfaces 1 to 2 times per day, avoiding aggravating factors (friction, heat, exfoliating manicures or pedicures), and wearing supportive shoes with thick cotton socks.

Clinical trials focused on the assessment and grading of PPE and evaluation of strategies used for management of PPE are needed.
